# Protocol, rationale and design of BE-PEOPLE (Bedaquiline enhanced exposure prophylaxis for LEprosy in the Comoros): a cluster randomized trial on effectiveness of rifampicin and bedaquiline as post-exposure prophylaxis of leprosy contacts

**DOI:** 10.1186/s12879-023-08290-0

**Published:** 2023-05-09

**Authors:** Assoumani Younoussa, Said Nourdine Samidine, Auke T. Bergeman, Alberto Piubello, Nissad Attoumani, Silahi Halifa Grillone, Sofie Marijke Braet, Achilleas Tsoumanis, Abdallah Baco, Aboubacar Mzembaba, Zahara Salim, Mohamed Amidy, Saverio Grillone, Rian Snijders, Paul Corstjens, Nimer Ortuno-Gutierrez, Carolien Hoof, Annemieke Geluk, Bouke C. de Jong, Epco Hasker

**Affiliations:** 1 National Tuberculosis and Leprosy control Program, The Union of Comoros, Moroni, Comoros; 2 Amsterdam University Medical Center, Amsterdam, The Netherlands; 3 Damien Foundation, Brussels, Belgium; 4 Institute of Tropical Medicine, Antwerp, Belgium; 5 Leiden University Medical Center, Leiden, the Netherlands; 6 Programs Department, Damien Foundation, Brussels, Belgium

**Keywords:** Leprosy, Single dose rifampicin, Bedaquiline, Surveillance, Post exposure Prophylaxis, (AM)resistance

## Abstract

**Background:**

Leprosy is an ancient infectious disease with an annual global incidence of around 200,000 over the past decade. Since 2018, the World Health Organization (WHO) recommends single-dose rifampicin as post-exposure prophylaxis (SDR-PEP) for contacts of leprosy patients. The Post ExpOsure Prophylaxis for Leprosy (PEOPLE) trial evaluated PEP with a double dose of rifampicin in Comoros and Madagascar. Preliminary results of this trial show some reduction in leprosy incidence in intervention villages but a stronger regimen may be beneficial. The objective of the current Bedaquiline Enhanced ExpOsure Prophylaxis for LEprosy trial (BE-PEOPLE) is to explore effectiveness of a combination of bedaquiline and rifampicin as PEP.

**Methods:**

BE-PEOPLE is a cluster-randomized trial in which 44 clusters in Comoros will be randomized to two study arms. Door-to-door screening will be conducted annually during four years, leprosy patients identified will be offered standard of care treatment. Based on study arm, contacts aged five years and above and living within a 100-meter radius of an index case will either receive bedaquiline (400-800 mg) and rifampicin (150-600 mg) or only rifampicin (150–600 mg). Contacts aged two to four years will receive rifampicin only. Household contacts randomized to the bedaquiline plus rifampicin arm will receive a second dose four weeks later. Incidence rate ratios of leprosy comparing contacts who received either of the PEP regimens will be the primary outcome. We will monitor resistance to rifampicin and/or bedaquiline through molecular surveillance in all incident tuberculosis and leprosy patients nationwide. At the end of the study, we will assess anti-*M. leprae* PGL-I IgM seropositivity as a proxy for the population burden of *M. leprae* infection in 8 villages (17,000 individuals) that were surveyed earlier as part of the PEOPLE trial.

**Discussion:**

The COLEP trial on PEP in Bangladesh documented a reduction of 57% in incidence of leprosy among contacts treated with SDR-PEP after two years, which led to the WHO recommendation of SDR-PEP. Preliminary results of the PEOPLE trial show a lesser reduction in incidence. The BE-PEOPLE trial will explore whether reinforcing SDR-PEP with bedaquiline increases effectiveness and more rapidly reduces the incidence of leprosy, compared to SDR-PEP alone.

**Trial registration:**

NCT05597280. Protocol version 5.0 on 28 October 2022.

## Background

BE-PEOPLE is a randomized controlled trial assessing the effectiveness of bedaquiline and rifampicin as post-exposure prophylaxis (PEP) for leprosy in Comoros. Leprosy is an ancient chronic infectious disease caused by *Mycobacterium leprae* or *M. lepromatosis* that is transmitted between humans, probably through the air, provoking dermatologic and neurologic manifestations in a subset of infected individuals, after long asymptomatic periods [1–3]. Delayed treatment initiation and/or inadequate prevention and management of complications may cause permanent disabilities, leading to stigma and discrimination [4]. During the incubation period, progression to leprosy disease might be prevented with post-exposure prophylaxis (PEP) [5]

In 2000, the World Health Organization (WHO) declared leprosy eliminated as public health problem based on a prevalence below 1 per 10,000 worldwide [6], assuming that after reaching these levels transmission of *M. leprae* will eventually cease. Leprosy global prevalence decreased from more than 5 million in the 1980s to 133,802 in 2021 [7]. However changes in case definition and reduction of treatment duration substantially, and negative effects of the Covid-19 pandemic contributed to decreasing registered global prevalence. Since 2006, leprosy incidence has plateaued at a level of above 200,000 cases annually, unveiling uninterrupted transmission of *M. leprae* [8]. To overcome the current stalemate, WHO endorsed the use of post-exposure prophylaxis with single-dose rifampicin (SDR-PEP) for contacts of leprosy patients [9, 10]. This was primarily based on the COLEP trial in Bangladesh, which documented a 57% reduction of leprosy incidence in contacts treated with SDR-PEP over a two years follow-up period [11]

The BE-PEOPLE trial described in this manuscript is a sequel of the PEOPLE trial conducted between 2019 and 2023 in Comoros and Madagascar. In PEOPLE, three modalities of SDR-PEP were compared to a control arm (arm 1), in which annual door-to-door case finding was conducted yet no PEP provided. Randomization was at the village level. The dosage of SDR-PEP used in the PEOPLE trial was double the WHO-recommended dose, i.e. 20 mg/kg. In arm 2 villages only household contacts received PEP. In arm 3 villages blanket PEP coverage was provided to anyone living within a 100-meter of an index case or to the entire village if more than 50% of the village population were included in this perimeter. In arm 4 villages, screening was accompanied by a serosurvey based on anti-PGL-I IgM (the first serosurvey with UCP-LFA on such a large scale in the field), an antibody marker of infection with *M. leprae* [12]. SDR-PEP was provided to all arm 4 household contacts as well as to anti-PGL-I IgM seropositive neighborhood contacts living within 100 m of any incident leprosy case, or anywhere in the village if more than 75% were living within the 100-meter perimeter [13]. The study is now in its final phase, screening, and data collection have been concluded. Preliminary analysis shows some protective effect of SDR-PEP but less than the 57% documented in the COLEP study. In the villages included in the PEOPLE trial in Comoros we found a continued high incidence of leprosy (approximately 1.1 per 1,000 per year) at the end of the study, incidence in Madagascar was very low in all study arms. For this reason, the BE-PEOPLE trial will take place in Comoros only. The aim of the trial is to compare effectiveness of a PEP regimen based on a combination of rifampicin and bedaquiline (BE-PEP) to that of the standard SDR-PEP regimen.

BE-PEOPLE was preceded by a phase 2 study to confirm the safety of BE-PEP. For this purpose, a leprosy endemic village that had been part of arm 1 of the PEOPLE trial was selected. In May 2022, the entire population of approximately 900 was screened for leprosy. After the screening, 300 eligible participants divided over three age brackets were randomized to either BE-PEP (rifampicin 600 mg + bedaquiline 800 mg for adults) or SDR-PEP (rifampicin 600 mg for adults) in an age de-escalating design. All participants had an ECG recorded pre-treatment and one day after treatment, and venous blood samples were collected pre-treatment and 14 days after treatment to be tested for liver functions (Aspartate aminotransferase AST/Alanine aminotransferase ALT). No major safety concerns emerged and the independent data safety and management board gave the green light for proceeding to phase 3, which is the subject of the current manuscript.

## Methods/design

### Objectives and hypothesis

The primary objective of BE-PEOPLE will be to compare effectiveness of BE-PEP to that of SDR-PEP at individual level. As a secondary objective, we will compare overall leprosy incidence between villages randomized to BE-PEP and villages randomized to SDR-PEP. Adverse events will be closely monitored and quantified per study arm.

Other objectives are to quantitatively assess anti-PGL-I IgM seropositivity in the population as a proxy for the population burden of *M. leprae* infection, and use seroprevalence as an indirect tool to monitor transmission in the area. The effect of all measures implemented (door-to-door screening, with or without PEP) on transmission will be assessed by comparing seroprevalence in 2019 to that in 2026 for villages that were in the original arm 4 of the PEOPLE trial and were also included in BE-PEOPLE. Thus, we aim to evaluate anti-PGL-I IgM serology as a surveillance tool that may be more sensitive than case based surveillance. Additionally, we will monitor the frequency of adverse events, rifampicin and bedaquiline resistance among leprosy and tuberculosis patients, nationwide and throughout the trial period. Finally, we will evaluate the cost by study arm, and cost-effectiveness of BE-PEP compared to SDR-PEP if relevant.

### Study design

The BE-PEOPLE trial is a cluster randomized trial in which 44 clusters from the islands of Anjouan and Mohéli (Comoros) will be randomized to two study arms. These include 34 out of 48 villages that were also part of the PEOPLE trial as well as nine new villages of which one has been divided into two. For randomization, clusters will be listed in order of decreasing baseline leprosy prevalence by island and by former PEOPLE trial study arm. They will then be randomized to arm 1 (BE-PEP), intervention, or arm 2 (SDR-PEP), comparator arm, of BE-PEOPLE. Participants residing in villages arm 1 who are between two and five years of age and with a weight of 10–20 kg are not eligible for BE-PEP but will be offered SDR-PEP instead.

A first round of PEP will be provided in 2023, within one month following the screening in each village. This will be considered the start of follow-up. Screening will take place on an annual basis until the fourth and final round in 2026, which will be the end of follow-up. Leprosy incidence will be measured at the individual level among those that received either SDR-PEP or BE-PEP, excluding those below five years of age or weighing less than 20 kg because they are not eligible for BE-PEP. We will also compare incidence at village level between arm 1 and arm 2 villages, irrespective of whether or not an individual received PEP and of the kind of PEP received.

Each individual provided PEP will be revisited the day after PEP intake to document any adverse events.

Throughout the study skin biopsies will be collected of all consenting leprosy patients and slit skin smears of multibacillary patients, as well as sputum samples of all consenting TB patients. In addition nasal and tongue swabs, tongue scraping, and face mask samples will be collected from both TB and leprosy patients. Those that are PCR-positive for *M. leprae*, and have sufficient amount of bacteria will be tested for resistance to the study drugs based on molecular methods.

Cost data will be collected alongside the trial.

Immediately following the final survey round in 2026, a serosurvey quantitatively assessing anti-PGL-I IgM antibodies will be conducted in BE-PEOPLE villages that were previously (since 2019) part of study arm 4 of the PEOPLE trial.

### Setting

The Union of Comoros is an archipelago in the Indian Ocean, north of Madagascar, and includes the islands Grand Comore (with the capital Moroni), Anjouan, and Mohéli. Since 2011, around 400 new leprosy cases are notified annually. The vast majority of these cases come from Anjouan and Mohéli with an estimated population of 450,000, equivalent to 888 new leprosy cases/ 1 million population per year.

The National Tuberculosis and Leprosy program strictly implements the strategies recommended by WHO, ensuring early diagnosis. Less than 3% of new leprosy cases have visible deformities and treatment completion rates are above 85% for both multibacillary (MB) and paucibacillary (PB) cases [7]. For decades enhanced case finding, including a camp approach, was combined with passive case finding. Since the PEOPLE trial started, annual door-to-door screening for leprosy was implemented in 48 villages [13]. Out of 1030 leprosy patients enrolled in a preceding study and in a sub-study of the PEOPLE trial between July 1, 2017, and Dec 31, 2020, 73.3% were positive for *M. leprae* by repetitive element-quantitative PCR (qPCR), illustrating the reliability of diagnoses made by the field teams. The same study analyzed resistance to rifampicin, fluoroquinolones, and dapsone, and found full susceptibility to all three drugs [14]. Although early case finding is ensured, combined with excellent treatment completion rates, transmission remains high as also illustrated by the 35% proportion of children under 15 years of age among 239 new patients diagnosed in 2021 [7].

In contrast with leprosy, tuberculosis (TB) in Comoros is mainly found on the main island, Grande Comore, contributing around 150 new TB cases notified annually [15]. In 2021, nationwide treatment coverage was 47% of the WHO estimated incidence of 35/100,000 population. The same year, treatment success was 92% for new TB cases, and 100% for previously treated and for those coinfected with HIV. Until now, no rifampicin-resistant TB has been notified in Comoros [16].

### Participants

Participants will be enrolled from 44 clusters: 34 in Anjouan and 10 in Mohéli. We will screen for leprosy including all residents and all ages, whenever leprosy is diagnosed treatment will be provided according to national guidelines. At baseline, all permanent residents aged two years or above, living within 100 m of an index case diagnosed during the period of 2018–2023, will be eligible for PEP. If this entails more than 50% of the population, the entire village population will be eligible for PEP. In arm 1 the BE-PEP regimen will be offered, except for participants not eligible because of age (below five years) and/or weight (below 20 kg) criteria. Such individuals may still receive SDR-PEP if otherwise eligible. In both arms, the exclusion criteria include cough of more than two weeks’ duration (presumptive pulmonary TB), signs of extrapulmonary TB, self-reported pregnancy or breastfeeding, antecedents of liver or kidney disease (clinically or documented by lab tests), allergy to rifampicin, and treatment with rifampicin in the last two years. For arm 1 there will be some additional criteria which include use of medications in the three weeks preceding PEP that are not included in the safe list for bedaquiline.

### Randomization

The 44 study clusters will be grouped into 10 categories, by island (Anjouan and Mohéli) and in relation to the PEOPLE trial (former arm 1,2,3, or 4, or new). Within each group, they will be ordered by decreasing baseline prevalence and pairs will be constituted of successive clusters. Within each pair, one will be randomized to arm 1, and the other to arm 2. An independent sponsor biostatistician will prepare the randomization schedule using SAS 9.4 (SAS Institute, Cary NC).

### Outcome measures

The primary outcome will be the leprosy incidence rate ratio between contacts who received SDR-PEP and contacts who received BE-PEP, excluding those in arm 1 who received SDR-PEP because of not being eligible for BE-PEP due to age and/or weight restrictions. In a secondary analysis, we will calculate the incidence rate ratios at the village level between arms 1 and 2, including all participants irrespective of whether or not they received PEP.

Other outcome measures will be the frequency of adverse events by PEP regimen and costing of both study arms, and serosurvey in 2026 quantitatively assessing anti-PLG-I IgM antibodies in a subset of villages. We will also report proportions of leprosy or TB patients with resistance to either of the study drugs and assess any trends if present. A cost-effectiveness of BE-PEP compared to SDR-PEP will be done if BE-PEP is shown to be more effective.

### Intervention implementation and data collection

Before the study begins, village elders will be informed about study objectives and procedures, followed by community sensitization. Annual door-to-door screening for leprosy will be conducted from 2023 to 2026, by teams that consist of experienced health services staff and community volunteers. Data to be collected include geographic coordinates of all households visited as well as individual data, in particular demographic data and for those present during the visit, health, and medical data, results of examinations for leprosy and/or TB, eligibility for PEP, acceptance/refusal of PEP, and occurrence of adverse events. Data entry will be performed directly in the field, making use of an Android app in REDCap (Research Electronic Data Capture). REDCap is widely used in research and is compliant with standards and applicable regulations of good clinical practice (GCP). Data can be entered in REDCap offline, to be uploaded to a secure server whenever internet connection is available. We will also make use of a paper form, one form per household, to record name, age, and gender of each individual enrolled. These forms have a pre-printed unique barcode for each individual recorded as well as a unique household ID. They will be entered in an MS Access database, from which new forms with household ID, names, ages, and barcodes can be printed each time a new survey round starts. The field staff will use these forms to find back households previously visited. When entering data in the REDCap forms, they will use these barcodes and household IDs, no other personal identifiers will be recorded.

TB patients diagnosed nationwide will be asked to sign an informed consent and will have sputum, nasal and tongue swabs, tongue scraping, and face mask sampling for genotyping of *M. tuberculosis* (MTB) DNA with the extended version of Deeplex MycTB that will include targets associated with rifampicin- and bedaquiline resistance. Target deep sequencing allows to detect early signs of resistance, as in minority mutant populations as little as 3% of the total bacterial population is present in the sample of a patient. Whole genome sequencing (WGS) may be used to track the dissemination of drug-resistant MTB. TB patients will be treated as per national guidelines.

The diagnosis of leprosy will be following WHO guidelines, based on three cardinal signs: patch with loss of sensation, enlarged peripheral nerves, and/or slit-skin smear (SSS) positive for acid-fast bacilli. All leprosy cases diagnosed will be confirmed by experienced national leprosy control program health staff. All new leprosy cases detected will be treated according to the national guidelines. Other skin diseases such as mycoses, scabies, or eczema will also be treated free of charge. Subject to informed consent, all incident leprosy patients in Anjouan and Mohéli will be enrolled in a sub-study in which slit skin smears, nasal and tongue swabs, tongue scraping, facial mask sampling, and skin biopsies from non-facial lesions will be sampled to be tested with quantitative polymerase chain reaction (qPCR). If sufficient DNA is available, we will conduct further molecular tests for *Mycobacterium leprae* using Deeplex-MycLep, and whole genome sequencing (WGS) to detect resistance to rifampicin, fluoroquinolone, dapsone, and bedaquiline, as well as to determine the genotype of circulating strains for transmission tracking.

During the first round of annual door-to-door screening for leprosy, residents will be informed about objectives and study procedures, and requested to provide written informed consent. They will then be individually assessed for PEP eligibility. Upon completion of screening in a village, a PEP eligibility zone will be determined based on presence of leprosy index cases within 100 m. If more than 50% of the population lives within a 100-meter radius of an index case, the entire village will be eligible. Then, provision of PEP will be organized according to the study arms in another visit. All new leprosy cases diagnosed in between annual screening rounds will also be taken into account in the study. Eligible contacts of these patients will receive PEP during the next round of PEP administration.

All participants who received PEP will be re-visited the next day for assessing vomiting and (serious) adverse events ([S]AE). Vomiting will be recorded but the dose will not be repeated. In case of SAE, the health worker will record the event and report it within 24 h to the sponsor, refer the patient for appropriate care, and follow up until resolution. Household contacts provided BE-PEP will be offered a second dose four weeks later, as preliminary data from the PEOPLE trial documented that their residual risk is still up to three times higher compared to the rest of the population. SDR-PEP will be offered only once. In addition, there will be passive reporting of adverse events over the period of up to 30 days post-PEP administration, afterwards only adverse events that are linked to the PEP intake will be documented. Pregnancy is an exclusion criterion but in case an existing pregnancy at the time of PEP administration only becomes apparent afterwards, the participant will be followed up until the time of delivery.

### Post-exposure prophylaxis

#### Single dose rifampicin

A single dose of rifampicin at 10 mg/kg is the current standard for PEP in leprosy for contacts aged two years and above, as per the 2018 WHO guidelines [17]. In the PEOPLE trial, rifampicin was used at 20 mg/kg as ‘Single Double Dose Rifampicin Post Exposure Prophylaxis’ or ‘SDDR-PEP’ [13] with no serious adverse events observed. However, in the BE-PEOPLE trial, we opted for using the WHO-recommended dosage of 10 mg/kg as a comparator arm to assess the effectiveness of bedaquiline added to the WHO-recommended PEP.

#### Rationale for using serology

Although detection of *M. leprae* infection remains a challenge in asymptomatic individuals, the presence of antibodies specific for phenolic glycolipid-I (PGL-I) correlates with the bacterial load [18]. Therefore, serosurveillance utilizing field-friendly tests detecting anti-PGL-I IgM antibodies can be applied to study (reduction of) the population burden of *M. leprae*, an indirect measure of the (reduction in) transmission as a result of the combination of early case finding through door-to-door screening, and PEP reducing progression to incident leprosy [19, 20].

### Rationale bedaquiline and rifampicin combination

Bedaquiline, the first new drug to be developed against *M. tuberculosis* in 40 years, received conditional FDA approval in 2012 for treatment of multi-drug resistant tuberculosis. Bedaquiline targets the subunit c of the ATP synthase in the respiratory chain and has become a ‘game changer’ in the treatment of TB patients with advanced resistance. Bedaquiline is typically given once daily for 2 weeks, followed by 3x weekly dosing for 6 months or longer. It has a very long half-life of around 7 months once steady state is established. In the clinical studies preceding bedaquiline approval, safety was established for a single supratherapeutic dose of 800 mg in healthy volunteers. In bedaquiline dose-ranging studies in TB patients, the highest dose used consisted of a loading dose of 700 mg bedaquiline followed by a dose of 500 mg on day 2, which showed the strongest early bactericidal activity [21]. In vitro *M. tuberculosis* studies show that bedaquiline as well as rifampicin are active against both replicating and non-replicating bacteria, whereas moxifloxacin and isoniazid only kill replicating bacteria [22]. The combination of rifabutin, with the same mode of action as rifampicin, plus bedaquiline produced sustained intracellular mycobactericidal activity that was greater than the sum of their individual effects [23]. While bedaquiline is a substrate of the cytochrome P450 isoenzyme CYP3A4, of which rifampicin is a strong inducer, a single dose as used for PEP is not expected to lead to drug-drug interactions [24].

The bioavailability of bedaquiline increases with food (a 2-fold increase in AUC, see label). The exposure of rifampicin, when administered with food, decreases slightly but this is not considered clinically relevant. Therefore, we will offer a snack when PEP is administered.

### Data analysis

To assess the effectiveness of PEP at the individual level, we will fit a Poisson model adjusted for follow-up time as an offset term with the villages nested in islands as a random effect and type of PEP (BE-PEP or SDR-PEP) as an explanatory variable, with SDR-PEP as reference category. We will exclude participants below the age or weight limits for BE-PEP, even if they have received SDR-PEP. For each individual, follow-up time will start the day he or she was last examined before the first dose of PEP was administered and will end either at the time of their last survey visit or at the time the individual was diagnosed with leprosy. All incident leprosy cases diagnosed after the first door-to-door screening in 2023 until the final survey in 2026 will be considered. Participants lost to follow-up after their first intake of PEP will be excluded.

Using a similar Poisson model, we will calculate the incidence rate ratio of leprosy at the village level between arms 1 and 2, including all participants, irrespective of whether they received PEP and the regimen received. Follow-up time will start on the median date of first PEP administration in each village and end on the median date of the final survey round. For incident leprosy cases follow-up time will end on the date of diagnosis. Although villages will be randomized, we will explore baseline prevalence of leprosy as a potential confounder.

In the villages included in arm 4 of the PEOPLE trial, we will conduct an anti-PGL I survey in 2026 to compare the seroprevalence rates with those of 2019 in different age groups at the village and island level.

We will calculate costs per person screened for leprosy and the cost per person provided either SDR-PEP or BE-PEP. If BE-PEP is found to be more effective, then the cost-effectiveness analysis will be performed with the average cost per case of leprosy averted per arm and island. Also, incremental costs per person treated will be calculated using SDR-PEP as a baseline.

Finally, the prevalence of bedaquiline and/or rifampicin-resistant strains will be calculated per island. We will include in the numerator all leprosy or TB patients with resistance to rifampicin and/or bedaquiline, and all leprosy or TB patients tested in the denominator. Any apparent annual trends will be tested with chi-square for trend to assess statistical significance.

### Sample size

For sample size calculations we used the methodology described by Hayes and Bennet for cluster randomized trials [25]. The aim is to demonstrate a 50% reduction in risk of leprosy over 3 years for those who received BE-PEP compared to SDR-PEP. Based on data from the PEOPLE trial we assume a risk of 1.1 per 1,000 per year in the SDR-PEP arm, i.e. a cumulative incidence of 3.3 per 1,000 over the 3-year follow-up. For the PEOPLE trial, we calculated a coefficient of variation between clusters (κm) of 0.34, in the BE-PEOPLE trial we assume a slightly higher Km, 0.4. With a power of 90%, an average cluster size of 1,708, and α = 0.05, 22 clusters per study arm would be required, i.e. 75,152 subjects for the two arms combined. To recruit 75,152 eligible subjects we need to target a population of approximately 124,000 in total. Therefore, we selected 43 villages accounting for an estimated population of 124,035, of which one will be divided into two clusters. Of the 43 villages selected, 34 are already part of the PEOPLE trial with a population of approximately 73,000. In addition, we have selected 9 new villages with an estimated population of 51,000.

To assess the effect of BE-PEP at the village level, the entire population examined will be considered in the analysis. If incidence at the village level is 1.1 per 1,000 per year, as observed in the PEOPLE trial, and a 50% reduction is achieved with BE-PEP, the available sample size would provide a power of approximately 90% over a three-year follow-up period.

### Ethics

The study will be carried out according to the principles stated in the Declaration of Helsinki, Good Clinical Practice (GCP), General Data Protection Regulation (GDPR), and all applicable regulations and according to established international scientific standards. A yearly update on the status of the study will be provided as required.

In Comoros, the BE-PEOPLE trial was approved by ‘Comité National d’Ethique pour les Sciences de la Vie et de la Santé’ (CNESS) (Réf.N°23/03/CNESS/PR) as well as by the ‘Direction Générale de la Santé’ (Réf.N°23/33/MSSPSPG/DGS). Approval was also received from the Institutional Review Board (IRB) of the Institute of Tropical Medicine (ITM). In addition, the study has been approved by the Ethics Committee (EC) of the University of Antwerp Hospital in Antwerp.

The study protocol has been included in the Clinicaltrials.gov public registry (on 28 October 2022, https://clinicaltrials.gov/ct2/show/NCT05597280).

## Discussion

Post-exposure prophylaxis for leprosy based on a single dose of rifampicin (SDR-PEP) is a key intervention in the current WHO strategy ‘Towards zero leprosy’ [26]. One of the four strategic pillars is to ‘Scale up leprosy prevention alongside integrated active case detection’. This includes a scale-up of preventive chemotherapy. In a modeling study, assuming coverage of contact tracing and screening for leprosy of 90%, 22 years are needed to decrease leprosy incidence by 90% in 110 countries affected [27]. This modeling study used the effectiveness documented in the pivotal COLEP trial in Bangladesh, which achieved a 57% reduction of leprosy incidence in close contacts benefiting from SDR-PEP over two years of follow-up [11]. But effectiveness of SDR-PEP may vary between epidemiological settings. In an earlier study on hyperendemic islands in Indonesia, a blanket approach covering the entire island population resulted in 75% reduction in leprosy incidence, compared to an island where no PEP was provided. However, providing SDR-PEP to close contacts only had no effect on the incidence at the island level [28]. SDR-PEP administered to all household contacts in Morocco, a country with low leprosy prevalence, resulted in a 16% annual decline in leprosy nationwide from 2012 to 2017 [29]. In Comoros so far leprosy incidence has remained fairly stable, even in villages that during the PEOPLE trial received blanket coverage with SDR-PEP at twice the regular dose. There is thus an urgent need to explore alternative PEP regimens, which is what BE-PEOPLE will set out to do. On theoretical grounds, we expect a synergistic effect between bedaquiline and rifampicin, which we will now try to confirm in an intervention trial. For providing a prophylactic regimen to healthy individuals, a high safety threshold is required. We also cannot jeopardize the efficacy of bedaquiline against *M. tuberculosis*, as key drug in the treatment of multi drug-resistant tuberculosis. For these reasons, the BE-PEOPLE study has put in place, several safeguards. Ahead of the phase 3 intervention trial, a phase 2 safety study was conducted in Comoros, which did not reveal significant risks of toxicity after a single dose of BE-PEP. To detect even the slightest risk of resistance to either bedaquiline or rifampicin being introduced by the BE-PEOPLE study, we will monitor drug resistance in all incident tuberculosis and leprosy patients for the duration of the study.

As potential weaknesses in the planned design of BE-PEOPLE, we are mostly concerned about the impact of door-to-door screening and treatment of incident leprosy cases. These measures alone may already cause so much reduction of leprosy incidence that the added effect of PEP becomes hard to measure. However so far during the PEOPLE study, incidence remained high despite annual door-to-door screening in all villages included. Another potential weakness is that the high pill burden of BE-PEP, 8 tablets of bedaquiline plus 4 capsules of rifampicin for an adult, may negatively affect acceptability.

If indeed the combination of bedaquiline and rifampicin will have significant additional efficacy when compared to rifampicin only, in particular in a high incidence setting such as Comoros, this will have important implications for the strategy put in place to achieve zero leprosy. To provide optimal information for finetuning the ‘Towards zero leprosy’ strategy, we will not only assess the individual effect of BE-PEP but also the effect at village level, realizing that important segments of the population are either not eligible for BE-PEP or will not agree to prophylactic treatment. Another important element is careful documentation of any adverse events, which may have profound consequences when deciding whether or not to apply BE-PEP at even larger scale. Even if based on the results of the phase 2 study there appear to be no major concerns, data on a larger population are essential in any policy debate. Similar considerations apply to the potential risk of introducing antimicrobial resistance to the study drugs.

We will also conduct a costing analysis and if indeed BE-PEP is more effective than SDR-PEP, a cost-effectiveness analysis. Finally, we will evaluate serosurveys based on quantitative anti-PGL-I IgM as a proxy for population burden of infection as an alternative to case-based surveillance, which may become an important asset once leprosy incidence drops to levels at which stochasticity will make it very hard to discern any trends in transmission. For all these reasons we expect the BE-PEOPLE study to provide crucial information to guide the further elimination strategy for leprosy.

## Data Availability

The data supporting the findings of this publication will be retained at the Institute of Tropical Medicine, Antwerp, and will not be made openly accessible due to ethical and privacy concerns. Data can however be made available after approval of a motivated and written request to the Institute of Tropical Medicine at ITMresearchdataaccess@itg.be.

## List of Abbreviations

AE: Adverse Event
PGL-I: Phenolic glycolipid-I
AR: Adverse Reaction
BCG: Bacille de Calmette Guérin
DF: Damien Foundation
IRB: Institutional Review Board
ITM: Institute of Tropical Medicine
MB: Multi bacillary
PB: Paucibacillary
NTLCP: National Tuberculosis and Leprosy Control Program
PEOPLE: Post ExpOsure Prophylaxis for LEprosy in the Comoros and Madagascar
PEP: Post Exposure Prophylaxis
qPCR: Quantitative Polymerase Chain Reaction
REDCap: Research Electronic Data Capture
SAE: Serious Adverse Event
SDDR-PEP: Single Double Dose Rifampicin Post-Exposure Prophylaxis
SDR: Single Dose Rifampicin
SSS: Slit-skin smears
